# Can computer simulators accurately represent the pathophysiology of individual COPD patients?

**DOI:** 10.1186/s40635-014-0023-0

**Published:** 2014-09-20

**Authors:** Wenfei Wang, Anup Das, Tayyba Ali, Oanna Cole, Marc Chikhani, Mainul Haque, Jonathan G Hardman, Declan G Bates

**Affiliations:** School of Engineering, University of Warwick, Coventry, CV4 7AL UK; Anaesthesia & Critical Care Research Group, School of Medicine, University of Nottingham, Nottingham, NG7 2UH UK

**Keywords:** COPD, Computer simulation, Mechanical ventilation, Critical care medicine, Model matching, Global optimization

## Abstract

**Background:**

Computer simulation models could play a key role in developing novel therapeutic strategies for patients with chronic obstructive pulmonary disease (COPD) if they can be shown to accurately represent the pathophysiological characteristics of individual patients.

**Methods:**

We evaluated the capability of a computational simulator to reproduce the heterogeneous effects of COPD on alveolar mechanics as captured in a number of different patient datasets.

**Results:**

Our results show that accurately representing the pathophysiology of individual COPD patients necessitates the use of simulation models with large numbers (up to 200) of compartments for gas exchange. The tuning of such complex simulation models ‘by hand’ to match patient data is not feasible, and thus we present an automated approach based on the use of global optimization algorithms and high-performance computing. Using this approach, we are able to achieve extremely close matches between the simulator and a range of patient data including PaO_2_, PaCO_2_, pulmonary deadspace fraction, pulmonary shunt fraction, and ventilation/perfusion (V̇/Q) curves. Using the simulator, we computed combinations of ventilator settings that optimally manage the trade-off between ensuring adequate gas exchange and minimizing the risk of ventilator-associated lung injury for an individual COPD patient.

**Conclusions:**

Our results significantly strengthen the credibility of computer simulation models as research tools for the development of novel management protocols in COPD and other pulmonary disease states.

**Electronic supplementary material:**

The online version of this article (doi:10.1186/s40635-014-0023-0) contains supplementary material, which is available to authorized users.

## Background

Chronic obstructive pulmonary disease (COPD) is a leading cause of mortality and disability internationally and is predicted by 2020 to be the third most likely cause of death [1,2]. COPD is a progressive disease and is associated with increasing frequency and severity of exacerbations. Mechanical ventilation (MV), either invasive or non-invasive, may be a life-saving measure in managing respiratory failure due to an acute exacerbation of COPD [3]. However, mechanical ventilation is also associated with significant rates of morbidity and mortality. An improved understanding of the underlying pathophysiologic mechanisms of COPD is essential for the development of more effective and more individualized ventilation strategies for COPD patients.

Computer simulators that can accurately represent the particular disease state of an individual COPD patient could be an extremely valuable research tool for investigating the respiratory pathophysiology of COPD and predicting the effects of specific MV settings on the patient. Many researchers have worked on the development of physiological simulators, and various types of mathematical models have been proposed in the literature (e.g., [4–11]). However, these models generally employed only a very small number of compartments for gas exchange - in this paper, we show that such model cannot provide an accurate representation of the particular heterogeneous effects of COPD on alveolar mechanics. Also, most previous efforts to match physiological simulators to specific disease states have relied on manually manipulating the parameters in the simulator. Loeppky and co-workers [7] used a two-compartment model to match the COPD patient data generated from the multiple inert gas elimination technique (MIGET). Besides the use of a rather simplistic model, the study suffered from the use of only two parameters to represent V̇/Q mismatching; this is a significant limitation, since gas exchange in COPD patients is often characterized by different V̇/Q patterns with two or three modes [12]. In more sophisticated multi-compartmental models, matching has been achieved with some success by adjusting the resistance for 100 compartments manually [13], a challenging and time-consuming task. Indeed, it is obvious that manual matching is only practical for relatively simple models with a small set of adjustable parameters and a limited set of patient data for matching.

A small number of previous studies have investigated the use of numerical optimisation approaches for the model matching task; a tidally breathing model has been matched to MIGET measurements from emphysema and embolism patients using the interior-reflective Newton algorithm [14], and two and three compartmental models were matched to data from intensive care patients with acute lung injury (ALI) using Brent's method [15]. These studies again used rather simple models with only a few compartments, and both studies employed very simple optimization algorithms, for which the quality of the matching achieved depended completely on the initial values chosen for the optimisation parameters. Since there is very little information available about how to choose these initial estimates, the likelihood of such approaches finding the best possible match to patient data is very small. Moreover, due to the simplified nature of the models used, continuous V̇/Q distributions could not be produced, and thus, the model outputs can only be compared to patient data at two or three isolated points on the V̇/Q curve.

In this paper, we investigate the minimum level of complexity required in a computer simulation model in order to provide a representation of COPD pathophysiology that is accurate enough to allow the simulator to be used for studies on the design of novel therapeutic strategies for individual patients. In order to address this question, we employ a sophisticated simulation model of lung physiology that incorporates tidal ventilation, pulsatile pulmonary blood flow, hypoxic pulmonary vasoconstriction (HPV), a realistic and validated oxygen-hemoglobin model, and up to 200 individually configurable alveolar compartments. Our simulator has been developed over the past decade and has been used and validated in a number of previous studies [16,17,13]. In the present study, we couple this simulator to software implementing a global optimization algorithm [18], based on evolutionary principles (a *genetic algorithm*) which allows large numbers of model parameters to be simultaneously optimized in order to match the model outputs to detailed COPD patient data. An attractive feature of our approach from the point of view of clinical researchers and practitioners is that it can be highly automated, so that the user does not need to have detailed knowledge of the underlying algorithms but can use the software to fit models of varying complexity to different patient data sets. To illustrate how the proposed simulator could be used to investigate questions of clinical relevance, we present the results of an investigation into computing ventilator settings that optimally manage the trade-off between ensuring adequate gas exchange and minimizing the risk of ventilator-associated lung injury for an individual COPD patient.

## Methods

### The computational simulator

The simulation used in this study is a multi-compartmental computational model that uses an iterative technique to simulate integrated respiratory and cardiovascular pathophysiological scenarios [17,19,20]. A detailed description of the principles and mathematical equations underlying the computational model implemented in our simulator is provided in Additional file 1. In contrast to previous models of COPD pathophysiology that included only two or three alveolar compartments, our model allows the user to define the number of compartments (each with its own individual mechanical characteristics) to be implemented in the simulation. This allowed us to investigate in detail the relationship between the number of compartments in the model and its ability to match individual patient data. Each *i*th alveolar compartment has a unique and configurable bronchiolar resistance *R*_*B,i*_ [cmH_2_O∙s/l], pulmonary vascular resistance *R*_*V,i*_[dyn∙s/cm^5^], stiffness index *S*_*i*_ [cmH_2_O/ml^2^], and extrinsic pressure *P*_ext,i_ [cmH_2_O](giving 4×N adjustable parameters in total, where *N* is the number of compartments). The ability to adjust these parameters individually across up to 200 alveolar compartments allows the model to recreate the heterogeneous effects of COPD on the overall physiology of the lung. The model also includes specific equations to represent the effects of alveolar collapse, threshold opening pressure, alveolar stiffening, and airway obstruction. The net effect of these components of the simulation is that the defining, clinical features of COPD may be observed in the model: alveolar gas trapping (with intrinsic positive end-expiratory pressure (PEEP)), collapse-reopening of alveoli (with gradual reabsorption of trapped gas if reopening does not occur), limitation of expiratory flow, and increased functional residual capacity - see Additional file 1 for further details.

### Patient data

Two different sets of patient data from the literature are used in this study. The first dataset is from [21], where a 55-year-old patient is sedated and paralyzed, and relevant data for configuring the model and ventilator settings are shown in Table 1. For this case, we attempt to match the outputs of our simulator to the data reported for the following patient variables - PO_2_, PCO_2_, deadspace, shunt, mean and standard deviation of V̇ and Q, and ventilation-perfusion distribution across a number of ranges (see Table 2). The second set of patient data is from [12]. The 64-year-old patient is reported to be in a stable condition, and relevant model and ventilator configuration parameters are shown in Table 3. For this case, we attempt to match our model directly to patient V̇/Q curves generated via MIGET measurements.

**Table 1:** **Model configuration for the first patient dataset**

**Parameter**	**Value**
Respiratory frequency [bpm]	13
Tidal volume [ml]	590
FIO_2_	0.4
Inspiratory flow pattern	Constant flow
Cardiac output [l/min]	5.0
PEEP [cmH_2_O]	0
IE	1:3
RQ	0.8

**Table 2:** **Matched parameter values and the reference data for the first patient dataset**

**No.**	**Parameter**	**Data**	**Model outputs (** ***N*** **= number of compartments)**			
			***N*** **= 10**	***N*** **= 25**	***N*** **= 50**	***N*** **= 100**
1	PaO_2_ [mm Hg]	125.2	165.29	143.18	133.73	127.92
2	PaCO_2_ [mm Hg]	46	43.08	44.73	43.62	45.76
3	Dead space fraction	64.9	61.24	62.76	61.28	65.28
4	Shunt fraction	6.8	12.9	9.02	8.07	7.2
5	mean_ V̇	0.99	1	1	1	1
6	mean_Q	0.27	0.2	0.2	0.25	0.2
7	sd_V̇	1	0.92	1.15	0.92	1.15
8	sd_Q	1.34	1.38	1.38	1.38	1.38
9	0.1 < V̇/Q < 1, V̇	19.9	20.43	20.06	22.68	19.94
10	1 < V̇/Q < 10, V̇	14.3	19.69	16.57	16.47	14.48
11	0.01 < V̇/Q < 0.1, P	15.8	22.63	18.86	14.64	16.16
12	0.1< V̇/Q < 1, P	62.1	55.21	58.12	65.57	64.92
13	1 < V̇/Q < 10, P	10.9	11.05	11.58	10.63	10.56
	Total matching error		1.33	0.46	0.11	0.10
	Simulation time [h]		11	32	41	67

**Table 3:** **Model configuration for the second patient dataset**

**Parameter**	**Value**
Respiratory frequency [bpm]	16
Tidal volume [ml]	410
FiO_2_	0.21
Inspiratory flow pattern	Constant flow
Cardiac output [l/min]	3.4
PEEP [cmH_2_O]	0
IE	1:3
RQ	0.8

### Automated matching to patient data

Exacerbations of COPD are frequently associated with deterioration in gas exchange and associated hypoxemia. Unsurprisingly, increased inequality in V̇/Q relationships appears to be the major determinant of these changes [1]. Therefore, a key requirement for the simulation of COPD pathophysiology is the ability to accurately match the V̇/Q distributions seen in patient data. In our simulator, the V̇/Q distribution can be manipulated by adjusting the bronchiolar resistance and pulmonary vascular resistance, stiffness, and extrinsic pressure for each compartment in the model. For example, by increasing vascular resistance in a region of the lung, an area of relative dead space can be created. While all parameters can be manually adjusted, this becomes impractical as the number of compartments *N* in the model increases, since the total number of parameters is 4×N.

To address this issue, we formulate the model-matching problem as an optimization problem, where the difference between the model outputs and the data is captured in a cost function, and the model parameters that can be varied are the variables for the optimization problem. As mentioned above, two sets of patient data are considered in this study. For both cases, a primary focus is on accurately representing the imbalance in the V̇/Q distribution caused by the disease state. In the first dataset [21], the horizontal axis of the V̇/Q diagram is divided into several segments on which the percentage of V̇/Q indicated in the data will be matched. Other parameters that also need to be matched include PaO_2_, PaCO_2_, and mean and standard deviation of ventilation and perfusion. For the second case, the whole V̇/Q curve is considered (i.e., every point on the curve is to be matched). The matching error can then be defined based on these data, which is given as:

$$ {E}_T = {\displaystyle \sum_{i=1}^n}{E_i}^2 $$

where *E*_*T*_ is the total residual error representing the matching accuracy, ${E}_i=\frac{x_i-{x}_{i d}}{x_{i d}}$ is the error for parameter *i*, *x*_*i*_ is the model output value for parameter *i*, and *x*_*id*_ is the value of the data for parameter *i*.

**Figure 1:**
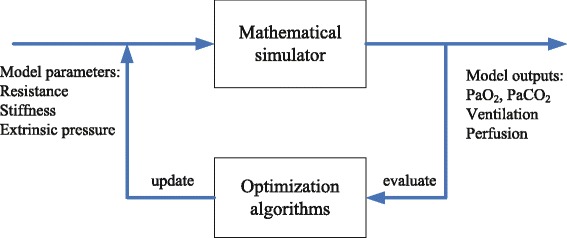
**Conceptual representation of the model matching process.**

Global optimization algorithms can then be used to find model parameter values that minimize the value of *E*_*T*_, i.e., minimize the difference between the model outputs and the data. The procedure is illustrated in Figure 1 - in each iteration, a set of parameter combinations are sent to the simulator, and the outputs from the simulator are evaluated by the optimization algorithm which then generates the updated parameter values for the next iteration until the termination criterion is reached (i.e., the condition to get a best matching is found).

In the model-matching procedure, the model parameters are allowed to vary continuously between physiologically realistic upper and lower bounds - see Table 4. COPD is characterized by restrictions to flow in airways (bronchi and bronchioles) due to increased excretion of mucus and inflammation of the bronchiolar walls, effectively reducing the radius of the tube through which the airways ventilate the alveoli. Based on the Hagen-Pouiselle relationship:

**Table 4:** **Model parameters – nominal values and allowable ranges (for** ***N*** **= 100)**

**Parameters**	**Nominal value**	**Variation ranges**
Bronchiolar resistance *R*_*Bi*_ [cmH_2_O∙s/l]	600	[300, 3 × 10^5^]
Vascular resistance *R*_*Vi*_ [dyn∙s/cm^5^]	1.6 × 10^4^	[8 × 10^3^, 8 × 10^6^]
Stiffness coefficient *S*_*i*_ [cmH_2_O/ml^2^]	0.05	[0.025, 0.15]
Extrinsic pressure *P*_ext,i_ [cm H_2_O]	28.8	[−20, 28.8]

$$ R\propto \frac{\eta L}{r^4} $$

the resistance (*R*) in a single tube is directly proportional to the length (*L*) of the tube and the viscosity (*η*) and inversely proportional to the fourth power of the radius (*r*^*4*^). To achieve the required precision in matching the extreme ranges of V̇/Q distributions for the second dataset, we have investigated the effect of increasing the upper bound on compartmental resistances from 500 to 2,000 times their nominal values, i.e., the radius of the airways can be reduced by as much as 85%. To ensure that this does not result in model configurations that produce unrealistic values for total lung resistance, we incorporated a constraint function into the optimization algorithm that limits the increase in the total lung inflow resistance to ten times its basic (healthy) value, as expected in patients with COPD [22].

Previous attempts to use optimization for matching models of pulmonary disease states to patient data have used rather simple algorithms that require good initial ‘guesses’ for the parameters in order to be effective. In the case of COPD, however, current understanding of the associated pathophysiology provides little guidance into how to choose initial values for the model parameters across large numbers of alveolar compartments. In this study, we therefore employed an advanced global optimisation algorithm known as a genetic algorithm. This general purpose stochastic search and optimization procedure, based on genetic and evolutionary principles [23], has been shown to have a much higher chance of finding optimal solutions for difficult problems with large numbers of variables. Full details of the particular optimization algorithm used in this study and how it was implemented with the model are provided in Additional file 1.

### Using the simulator for clinical investigations

To illustrate how the simulator can be used for clinical investigations, we consider the problem of identifying ventilator settings that minimize the risk of ventilator-associated lung injury (VALI). We consider the following five key ventilator settings as variable parameters that may be adjusted to optimize the trade-off between effective gas exchange and minimizing the risk of VALI: (1) tidal volume (Vtidal, [ml]) - the volume of air traveling in or out of the patient's lungs during every breath; (2) ventilation rate (VentRate, [breaths/min]) - the number of breaths per minute; (3) duty cycle (I:E) - the ratio of inspiratory time to total ventilatory cycle duration; (4) PEEP, [cmH_2_O] - the positive pressure in the lungs at the end of exhalation; and (5) fraction of inspired oxygen (FIO_2_) - the fraction of oxygen constituting the inhaled volume of gas as provided by the mechanical ventilator.

The maximum allowable ranges of variation for the values of these parameters have been defined based on current clinical practice and to be consistent with data available from clinical trials [24,25]. Vtidal is allowed to vary within a range from 390 to 650 ml, corresponding to 6 to 10 ml/kg for a body weight of 65 kg. VentRate is bounded within the range 9 to 16 breaths/min, I:E is limited to the interval 0.25 to 0.5 (i.e., a ratio between 1:4 and 1:2), PEEP is constrained within 0 to 5 cmH_2_O, and FIO_2_ is bounded within 0.21 to 1. A summary is provided in Table 5.

**Table 5:** **MV setting parameter variation bounds and desired model outputs**

	**Variation ranges**	
	**Acceptable values**	**Desired**
MV setting parameters		
Vt [ml]	[390, 650]	
VentRate [bpm]	[9,16]	
I:E	[0.25, 0.5]	
PEEP [cmH_2_O]	[0, 5]	
FiO_2_	[0.21, 1]	
Model outputs		
PO_2_ [kPa]	>8	12
PCO_2_ [kPa]	>4, <8	5.3
Palv [kPa]	<4	-

Three key physiological indicators are also defined. To monitor effective arterial oxygenation, partial pressure of oxygen, PaO_2_, needs to be considered. In order to maintain effective arterial oxygenation, PaO_2_ is constrained to be higher than 8 kPa, with a desired value of 12 kPa. Arterial partial pressure of carbon dioxide, PaCO_2_ is another key indicator of alveolar ventilation that also indirectly reflects acid-base balance. PaCO_2_ is constrained to be between 4 and 8 kPa with a desired value of 5.3 kPa. The risk of barotrauma is proportional to the peak alveolar pressure, (Palv, kPa above atmospheric pressure), and Palv is limited to 4 kPa, where Palv is calculated as the average of the peak pressure in the most highly pressurized 25% of all alveoli.

Requirements on the above physiological indicators can be captured as an optimization problem and formulated mathematically as:

$$ \min \left\{{J}_1,{J}_2\right\} $$

where

$$ {J}_1={w}_1\left|{\mathrm{PaO}}_2-12\right|+{w}_2\left|{\mathrm{PaCO}}_2-5.3\right| $$

$$ {J}_2={w}_3{P}_{alv} $$

Large values of *J*_1_ will be produced by ventilator settings that provide poor gas exchange, while large values of *J*_2_ will be produced by combinations of ventilator settings that cause high peak alveolar pressures (and hence increase the risk of VALI). By requiring both *J*_1_ and *J*_2_ to be minimized simultaneously, we can search for combinations of ventilator settings that optimally manage the trade-off between effective gas exchange and minimizing the risk of VALI. w_1_,w_2_, and w_3_ are weighting functions that are used in the optimization process to ensure that equal priority is given to each of the different objectives. Since we are trying to minimize two objectives *J*_1_ and *J*_2_ at the same time, a multi-objective optimization algorithm called non-dominated sorting genetic algorithm II (NSGA-II) was used here [26].

## Results

### Matching results for the first dataset

For the first dataset, the ventilation-perfusion distribution is characterized by several segments based on the V̇/Q ratio, which includes the amount of ventilation in the ranges 0.1< V̇/Q < 1 and 1< V̇/Q < 10, and the amount of perfusion in the ranges 0.01< V̇/Q < 0.1, 0.1< V̇/Q < 1, and 1< V̇/Q < 10 together with the shunt and dead space. Other data considered are the PO_2_, PCO_2_, mean V̇/Q ratio of the pulmonary blood flow, and ventilation distribution as well as standard deviations (dispersion) of pulmonary blood flow and ventilation. The simulation was performed separately for four cases with the total number of compartments in the model being varied between 10, 25, 50, and 100 to represent different levels of model complexity.

**Figure 2:**
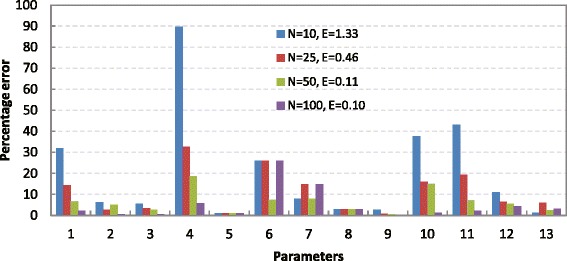
**Comparison of matching errors for different numbers of compartments.** Bars show matching error for each of the 13 parameters for simulation models with *N* = 10 (blue), 25 (red), 50 (green), and 100 (purple) compartments; E is total matching error.

Figure 2 and Table 2 report the fitted results for each scenario. It can be seen that incorporating a larger total number of compartments into the model significantly improves its ability to accurately represent the patient data, with the total fitting error reduced from 1.33 for 10 compartments to 0.46 for 25 compartments, to 0.11 for 50 compartments. Interestingly, when the total compartment number is increased from 50 to 100, the fitting error only reduces from 0.11 to 0.10, indicating that for this particular dataset, 50 compartments represent an adequate level of complexity. Considering that the computational burden associated with the parameter optimization rises proportionally with the complexity of the model, the ability to identify just how complex a model needs to be is clearly of primary importance for this type of problem.

### Matching results for the second dataset

**Figure 3:**
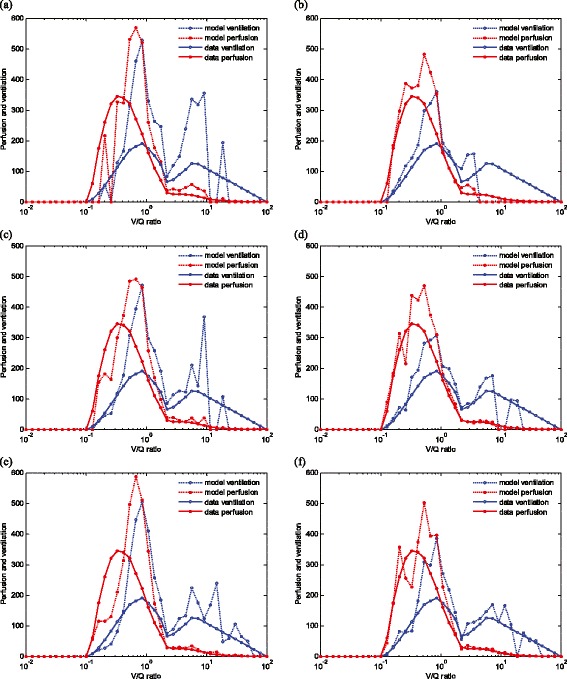
**Simulated V̇/Q distribution compared with the data.** *N* is the number of compartments used, ${\overline{R}}_{Bi}$ denotes upper bounds of bronchiolar resistance, and ${\overline{R}}_{Vi}$ denotes upper bounds of vascular resistances for the *i*th compartment. **(a)** *N* = 50, ${\overline{R}}_{Bi}$ = 3 × 10^5^, ${\overline{R}}_{Vi}$ = 8 × 10^6^, Error = 5.68. **(b)** *N* = 50, ${\overline{R}}_{Bi}$ = 1.2 × 10^6^, ${\overline{R}}_{Vi}$ = 3.2 × 10^7^, Error = 4.25. **(c)** *N* = 100, ${\overline{R}}_{Bi}$ = 3 × 10^5^, ${\overline{R}}_{Vi}$ = 8 × 10^6^, Error = 4.46. **(d)** *N* = 100, ${\overline{R}}_{Bi}$ = 1.2 × 10^6^, ${\overline{R}}_{Vi}$ = 3.2 × 10^7^, Error = 3.24. **(e)** *N* = 200, ${\overline{R}}_{Bi}$ = 3 × 10^5^, ${\overline{R}}_{Vi}$ = 8 × 10^6^, Error = 3.91. **(f)** *N* = 200, ${\overline{R}}_{Bi}$ = 1.2 × 10^6^, ${\overline{R}}_{Vi}$ = 3.2 × 10^7^, Error = 2.80.

In the second dataset, we attempt to reproduce in our model the complete V̇/Q curve generated from MIGET results [12]. Forty evenly distributed points are selected along the logarithm axis from 0.01 to 100 with each point representing an equal small segment. The ventilation and perfusion at each point are calculated by summing up the ventilation and perfusion of all alveoli with V̇/Q ratio that falls into the respective segment. The values at each point are then compared with the data, and the total error was calculated based on Equation 1. An initial minimum number of 50 compartments were used in the model, based on the results of the previous dataset reported above. However, as shown in Figure 3a, with this number of compartments, a large discrepancy is observed between the data and best model fit (total fitting error 5.68). When the number of compartments is increased to 100 and 200, however, it can be seen from Figure 3c,e that the ability of the model to represent the V̇/Q curve improves, with the fitting error reducing to 4.46 and 3.91, respectively. To improve the model fit over the left part of the V̇/Q curve, higher bounds on the resistance variation $\overline{R}$ were then allowed in the optimization procedure. Figure 3b shows that, for 50 compartments, the V̇/Q curve in the range of 0.1 to 3 is matched much more closely after using a higher upper bound for the resistance, and the total matching error E is reduced from 5.68 to 4.25. Increasing the number of compartments to 100 and 200 further reduces the matching error to 3.24 and 2.80, respectively, so that, as shown in Figure 3f, a very accurate representation of the total V̇/Q curve can be produced by the model. Note that, for the higher upper bound on the resistances, the use of a constraint function in the optimization algorithm to ensure that the total lung resistance stays within physiologically reasonable values for COPD patients results in a doubling of the overall computation time required for the optimization to converge.

### Computation of optimal ventilation strategies using the simulator

**Figure 4:**
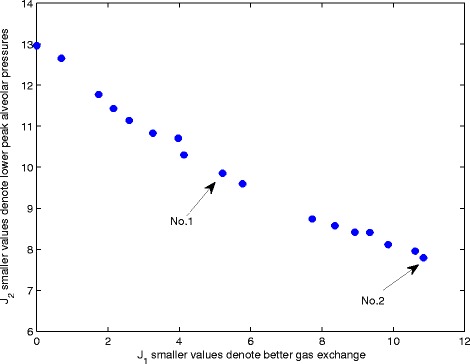
**Pareto front from the multi-objective optimization.** Shows the tradeoff between maximizing gas exchange and reducing the risk of VALI.

Table 6 compares the optimal MV settings computed using our simulator with those provided in the first patient dataset [21]. Figure 4 shows the so-called Pareto front returned by the NSGA-II algorithm. Each point on the Pareto front corresponds to a combination of ventilator settings for which any improvement in one objective function (e.g., gas exchange) necessarily results in a deterioration in the other (e.g., peak alveolar pressure). Thus, the Pareto front defines the trade-off inherent in the problem and allows us to investigate how different combinations of ventilator settings manage that trade-off. The optimal settings reported in Table 6 correspond to point No. 1 in Figure 4 and provide the most balanced solution to the two competing objectives. Compared with the data values, the peak alveolar pressure has been reduced from 5.1 kPa to 3.8 kPa, so that it is now within the maximum specified value of 4 kPa. This improvement has been achieved at the cost of a small increase in PaCO_2_ (from 6.1 to 6.3 kPa), while the value of PaO_2_ (12.2 kPa) is now very close to its desired value of 12 kPa. This ‘rebalancing’ of the ventilator settings in favor of a more lung protective strategy has been achieved principally by reducing tidal volume from 590 to 506 ml, while simultaneously increasing the ventilation rate from 13 to 15 bpm. Changes in the other three ventilator settings are more modest, indicating that they have a relatively smaller influence on the chosen physiological indicators for this patient. Moving along the curve shown in Figure 4, different combinations of ventilator settings are computed which place more emphasis on one objective or the other. Point No. 2, for example, corresponds to a maximally protective strategy, where Palv has been further reduced to 2.8 kPa, at the cost of increasing PaCO_2_ to 7.4 kPa and reducing PaO_2_ to 11.9 (note that both PaCO_2_ and PaO_2_ are still within their specified limits).

**Table 6:** **Optimal MV settings and model outputs compared with first patient dataset**

	**Data**	**Optimal**
MV setting parameters		
Vt [ml]	590	506
VentRate [bpm]	13	15
I:E	0.33	0.32
PEEP [cmH_2_O]	0	1
FiO_2_	0.4	0.3
Model outputs		
PaO_2_ [kPa]	17	12.2
PaCO_2_ [kPa]	6.1	6.3
Palv [kPa]	5.1	3.8

## Discussion and conclusions

The high mortality and morbidity associated with COPD will make the condition a critical health burden for the future, particularly if current trends are allowed to continue [27,28]. The main treatment option for critical exacerbations of COPD remains mechanical ventilation (non-invasive and invasive) and for long-term management, oxygen therapy [25]. Prognosis of COPD is worsened with underlying respiratory failures, adding greatly to the cost of treating the disease [29,30]. Research into the condition using animal models of lung injury and *in vitro* experiments have failed to show a clear way forward, and large-scale clinical trials to monitor long-term effects in this fragile population are not possible [31,6].

Computer simulations [6,32] offer a potentially powerful alternative platform for research into the development of new treatment strategies, but no previous studies have been able to demonstrate adequate matching to the particular disease characteristics of COPD. In this study, we have described a computer simulation model that can accurately simulate an individual COPD patient receiving MV. Our study has focused on single patient subjects described in the literature, and the two studies that have been selected represent the spectrum of COPD cases that are found in clinical practice. Our results clearly show the importance of employing mathematical models of sufficient complexity to allow an accurate representation of disease states in individual patients. In particular, the heterogeneous effects of COPD on lung alveoli in different regions of the lung make it essential that large numbers of compartments are included in the model - for all datasets considered, matching between the simulation outputs and patient data was significantly improved as the number of alveolar compartments in the model was increased.

The need for a large number of compartments with heterogeneous dynamics creates an additional challenge - how to choose appropriate values for the large number of corresponding model variables. Indeed, a second major conclusion of our study is that ‘manual’ tuning of such parameters is simply not feasible for models of the complexity required. Instead, we have shown how global optimization algorithms, implemented using sophisticated parallel processing protocols, can allow optimal values of very large numbers of parameters to be identified. A significant advantage of this approach is that it can be largely automated, with the clinical researcher needing only to input the patient data and the simulation platform taking care of all other aspects of the model-matching process.

Due to the nature of COPD, multiple comorbidities are associated with the symptoms and development of COPD including inflammation and heart and kidney dysfunctions. This presents a unique challenge to the clinician in terms of maintaining the correct balance between raising pressures and delivering oxygen, while keeping hypercapnea and hypoxia to a minimum [33,34]. The clinician needs to monitor multiple aspects of physiology simultaneously, and many adverse effects are not always immediately clear. Currently, MV is applied in a rather generic manner; however, the clinicians have recognized the need for accounting for inter-patient variation [35,36]. VALI due to overstretching of lung parenchyma can be minimized by applying lower tidal volumes [37], but this needs to be achieved without allowing PaCO_2_ and pH to rise to dangerous levels (permitted hypercapnea). Sophisticated computer simulation platforms that can accurately represent individual COPD patient characteristics could provide a powerful experimental tool for the development of improved treatment strategies for COPD that address many of the above issues.

## Additional file

Additional file 1:
**Simulation model description and optimization algorithm description.**
